# The impact of vector migration on the effectiveness of strategies to control gambiense human African trypanosomiasis

**DOI:** 10.1371/journal.pntd.0007903

**Published:** 2019-12-05

**Authors:** Martial L. Ndeffo-Mbah, Abhishek Pandey, Katherine E. Atkins, Serap Aksoy, Alison P. Galvani

**Affiliations:** 1 Department of Veterinary Integrative Biosciences, Texas A&M College of Veterinary Medicine and Biomedical Sciences, College Station, TX, United States of America; 2 Department of Epidemiology and Biostatistics, Texas A&M School of Public Health, College Station, TX, United States of America; 3 Center for Infectious Disease Modeling and Analysis, Yale School of Public Health, New Haven, CT, United States of America; 4 Department of Epidemiology and Microbial Diseases, Yale School of Public Health, New Haven, CT, United States of America; 5 Department of Infectious Disease Epidemiology, Faculty of Epidemiology and Population Health, London School of Hygiene and Tropical Medicine, London, United Kingdom; 6 Centre for Mathematical Modelling of Infectious Diseases, London School of Hygiene and Tropical Medicine, London, United Kingdom; 7 Centre for Global Health, The Usher Institute for Population Health Sciences and Informatics, Edinburgh Medical School, The University of Edinburgh, Edinburgh, United Kingdom; CIRAD, FRANCE

## Abstract

**Background:**

Several modeling studies have been undertaken to assess the feasibility of the WHO goal of eliminating gambiense human African trypanosomiasis (g-HAT) by 2030. However, these studies have generally overlooked the effect of vector migration on disease transmission and control. Here, we evaluated the impact of vector migration on the feasibility of interrupting transmission in different g-HAT foci.

**Methods:**

We developed a g-HAT transmission model of a single tsetse population cluster that accounts for migration of tsetse fly into this population. We used a model calibration approach to constrain g-HAT incidence to ranges expected for high, moderate and low transmission settings, respectively. We used the model to evaluate the effectiveness of current intervention measures, including medical intervention through enhanced screening and treatment, and vector control, for interrupting g-HAT transmission in disease foci under each transmission setting.

**Results:**

We showed that, in low transmission settings, under enhanced medical intervention alone, at least 70% treatment coverage is needed to interrupt g-HAT transmission within 10 years. In moderate transmission settings, a combination of medical intervention and a vector control measure with a daily tsetse mortality greater than 0.03 is required to achieve interruption of disease transmission within 10 years. In high transmission settings, interruption of disease transmission within 10 years requires a combination of at least 70% medical intervention coverage and at least 0.05 tsetse daily mortality rate from vector control. However, the probability of achieving elimination in high transmission settings decreases with an increased tsetse migration rate.

**Conclusion:**

Our results suggest that the WHO 2030 goal of G-HAT elimination is, at least in theory, achievable. But the presence of tsetse migration may reduce the probability of interrupting g-HAT transmission in moderate and high transmission foci. Therefore, optimal vector control programs should incorporate monitoring and controlling of vector density in buffer areas around foci of g-HAT control efforts.

## Author summary

Gambian human African trypanosomiasis (g-HAT), also known as sleeping sickness, is a vector-borne parasitic disease transmitted by tsetse flies. If untreated, g-HAT infection will usually result in death. Recently, the World Health Organization (WHO) has targeted g-HAT for elimination through achieving interruption of transmission by 2030. To help inform elimination efforts, mathematical models have been used to evaluate the feasibility of the WHO goals in different g-HAT transmission foci. However, these mathematical models have generally ignored the role that tsetse migration may have in the spread and reemergence of g-HAT. Using a mathematical model, we evaluate the impact of tsetse migration on the effectiveness of current intervention measures for achieving interruption of g-HAT transmission in different transmission foci. We consider different interventions such as enhanced screening and treatment and vector control. We show that vector control has a great potential for reducing transmission. Still, the presence and intensity of tsetse migration can undermine its effectiveness for interrupting disease transmission, especially in high transmission foci. Our results indicate the need of accounting for tsetse surveillance and migration data in designing vector control efforts for g-HAT elimination.

## Introduction

Human African trypanosomiasis (HAT), also known as sleeping sickness, is a vector-borne parasitic neglected tropical disease. HAT is endemic throughout Sub-Saharan Africa, resulting in significant morbidity and mortality among affected communities [1]. There are two forms of HAT infections caused by two subspecies of the *Trypanosoma brucei (Tb*.*)* parasite, *T*.*b*. *gambiense* (*Tbg*) and *T*.*b*. *rhodesiense* (*Tbr*) [2]. The gambiense form of HAT (g-HAT), caused by *Tbg*, is a slow-progressing disease endemic in West and Central Africa. The Rhodesiense form of HAT, caused by *Tbr*, is acute fast-progressing disease which is endemic in East and Southern Africa [1]. HAT is transmitted by different tsetse fly species which are classified as forest, riverine, or savannah species, according to their habitat preference [2]. Riverine species, with habitat generally consisting of strips of woodland bordering rivers or lakes, are the main vector of *Tbg* [3,4].

The 2012 London Declaration on Neglected Tropical Diseases targeted g-HAT for elimination as a public health problem by 2020 [5]. This 2020 goal is defined by the WHO as a 90% reduction of annual disease incidence in areas reporting more than 1 case per 10,000 inhabitants compared to 2000–2004, and fewer than 2000 annually reported cases across the whole of Africa [5]. A more ambitious goal of complete interruption of g-HAT transmission was also set for 2030. Control efforts for g-HAT elimination have largely hinged on treatment through passive and active case detection [6,7]. Recently developed vector control measures, such as tiny targets and sterile insect technique, have been shown to be highly effective methods to reduce tsetse populations and to control HAT in some endemic foci [8–12].

Though significant progress toward g-HAT elimination as a public health problem has been made in many foci across Africa, additional efforts are required to achieve full interruption of g-HAT transmission [5]. Previous mathematical models have been developed to evaluate the feasibility of vector control and medical intervention strategies for achieving g-HAT elimination in different foci across sub-Saharan Africa [11,13–21]. With only a few exceptions [18,19], most of these models have generally considered tsetse within HAT foci as an isolated population surrounded by areas unsuitable for tsetse. Though this may be the case for some small and locally isolated settings, it is not the case for most g-HAT foci, which are generally small foci where tsetse populations may immigrate from surrounding HAT-free areas [22–25]. This is specifically important as vector control operations for g-HAT are generally conducted serially within small targeted regions located within larger tsetse-dense area [10,26,27]. In this case, vector-controlled areas are at risk of being re-populated through migration from neighboring uncontrolled areas. Hence this knowledge is important in defining the scale vector control needs to be applied at to interrupt transmission.

We developed a dynamic model of g-HAT transmission in a single human and tsetse populations, accounting for tsetse fly migration from neighboring tsetse population. We considered different endemic HAT transmission intensity settings representative of high, moderate, and low transmission intensity. We used the model to evaluate the impact of tsetse migration rate on the feasibility of interrupting g-HAT transmission using intervention measures currently used in g-HAT control: enhanced case detection/screening and treatment and vector control using insecticide treated targets or traps.

## Methods

### Model

We developed a mathematical model for g-HAT transmission in an endemic focus accounting for tsetse fly migration into the tsetse cluster population. The HAT transmission model was a deterministic vector-host with a Susceptible, Exposed, Infectious, Recovered (SEIR) model for the vector and a Susceptible, Exposed, Infectious, Treated (SEIT) model for the host. Following previous HAT models [14,28,29], we categorized humans and tsetse according to their disease status: susceptible (*H*_*S*_, *V*_*S*_), exposed (*H*_*E*_, *V*_*E*_), infectious (*H*_*I*_, *V*_*I*_), treated/recovered (*H*_*T*_, *V*_*R*_). To account for the high risk of tsetse flies infection during their first blood meal and their reduction of susceptibility with age (hours after eclosion of the fly from the puparium) at first meal [30–32], we assumed that tsetse are susceptible to trypanosome infection only during their first blood-meal and within 24 hours after emergence from pupa (*V*_*P*_) to the adult stage (*V*_*S*_) [30–32]. After their first feeding, susceptible tsetse (*V*_*S*_) either become infected by feeding on an infectious human and enter the exposed state (*V*_*E*_), or become recovered (*V*_*R*_). After incubation, exposed tsetse (*V*_*E*_) become infectious (*V*_*I*_) for the rest of their lives and can infect humans. For simplicity, we assumed that the g-HAT focus is surrounded by g-HAT free area uninhabited by humans, with tsetse flies migrating between g-HAT focus and the surrounding areas. Tsetse migration rate was informed by empirical estimates of 0.05 to 0.85 per generation [31,32]. Such a situation is typical of many HAT endemic rural settings in Western and Central Africa, where humans live within small villages surrounded by uninhabited areas suitable to tsetse.

We assumed heterogenous risk behavior across the human population with individuals divided into high and low risk groups. The high-risk group had a higher contact rate with tsetse than the low risk group. The high risk group may represent individuals involved in activities that require increased exposure to water sources or other tsetse dense areas [33]. Humans may acquire infection after a bite from an infectious tsetse (*V*_*I*_). Upon infection, humans become exposed and enter the infectious stage 1 of g-HAT (*H*_*I*_) after an incubation period. Infected humans progress from stage 1 to stage 2 of infections, characterized by the severity of disease symptoms. We assumed that stage 1 and stage 2 contributes equally to disease transmission. If untreated, infected people in stage 2 will die. Successfully treated g-HAT patients are temporarily both immune to reinfection and not exposed to tsetse bites because of hospitalization before returning to full susceptibility and likelihood of tsetse bite exposure (*H*_*S*_). Tsetse do not feed on humans only, but for simplicity, non-humans hosts were not explicitly included in our model. This was implicitly taken into account by assuming that only a fraction on tsetse bites were on humans. In addition, we assumed that non-human hosts are not a reservoir for g-HAT, given that this question has not yet been fully elucidated [34]. We provide a detailed description of our model in the Supplementary Materials.

### Model calibration

We calibrated our model to three transmission intensity settings, representing high, moderate, and low g-HAT incidence foci. Transmission intensity was defined as 1 to 10 cases per 100,000 annually for low incidence settings, 10 to 100 cases per 100,000 annually for moderate incidence, and 100 to 1,000 cases per 100,000 annually for high incidence [5]. We assumed infection dynamics are at equilibrium under a long-standing screen and treat program with 40% coverage [35,36]. We calibrated the model independently to the three incidence ranges: low, moderate and high transmission settings. Model calibration was performed using a Bayesian Markov Chain Monte Carlo (MCMC) approach [37,38], with prior parameter distributions obtained from published literature (Supplementary Materials). We used the Metropolis-Hastings algorithm [38], and convergence of iterative chains was assessed using the Brooks-Gelman-Rubin diagnostic criterion [39].

### Intervention strategies

Medical intervention through staging and treatment of infected individuals has long been the pillar of g-HAT control efforts [5]. This intervention hinges on identifying/screening and treating disease cases; and thereby reducing disease burden and risk of new infection within a community. Here, we assume that screen and treat operates by removing stage 1 and stage 2 cases at a daily rate. We relate annual screen and treat coverage *r* to the daily screen and treat rate by *γ_T_* = −*ln*(1−*r*/100)/365 [18]. The baseline screen and treat coverage was set to 40% annual coverage. We assumed screen and treat efforts could be enhanced by targeted and intensified screening. Intensified screening can be achieved through increased frequency and coverage of active case finding and treatment and increased access to g-HAT testing and treatment in local healthcare facilities. Targeted screening can be achieved by mobile healthcare worker teams, who find individuals living in affected areas and identify infection, targeting both high- and low-risk people equally. This approach increases surveillance coverage by providing house-to-house screening and improving access to hard to reach communities [40]. We assumed an equal coverage of low and high-risk group individuals. To account for variability in screen and treat coverage and compliance, we varied screen and treat coverage from 40–90%.

Vector control may be implemented through the use of insecticide treated targets or traps [41]. The effectiveness of traps or targets for reducing tsetse population depends on several factors such as tsetse species and environmental conditions [41,42]. Empirical studies have shown that highly effective vector control programs may reduce tsetse population by more than 90% after the first year [27,41,43]. We modeled vector control as an additional density-independent mortality rate of adult tsetse [41] which does not impact the tsetse population carrying capacity. For simplicity, we assume the daily rate is constant through time, but we varied this daily rate from 0.01–0.08 [41]. We evaluated a wide range of possible intervention strategies combining enhanced screen and treat with vector control efforts.

### Forward projections

The model was coded using MATLAB R2018b and differential equations were solved using the ode45 solver which is based on an explicit Runge-Kutta method [44]. We initiated the model with the tsetse population set to its asymptotic equilibrium in the absence of vector control and g-HAT transmission. We seeded infection in the human population with a low prevalence of 10^−5^ in the infectious stage 1 compartment of the high-risk group. For simplicity, we assumed that the screen and treat intervention was implemented at its baseline value of 40% annual coverage from year 1. The model was run for 200 years to ensure that it has reached its asymptotic state. Though the g-HAT equilibrium point was reached within 50 years for many model’s input parameters values, a longer time period was required for the system to reach equilibrium for some parameter values.

From the g-HAT equilibrium, the model was run for 30 years to evaluate the effectiveness of enhanced screen and treat intervention and vector control for interrupting g-HAT transmission in disease foci for each of the three transmission intensity settings. We evaluated the probability of interrupting disease transmission as the proportion of samples from our posterior distributions for which annual disease incidence was lower than 10^−7^ from that year onwards. Assuming that enhanced medical intervention and vector control are initiated in 2020, a 10-year mark for g-HAT elimination would be equivalent to reaching the WHO 2030 goal.

## Results

### Medical intervention without vector control

**Fig 1:**
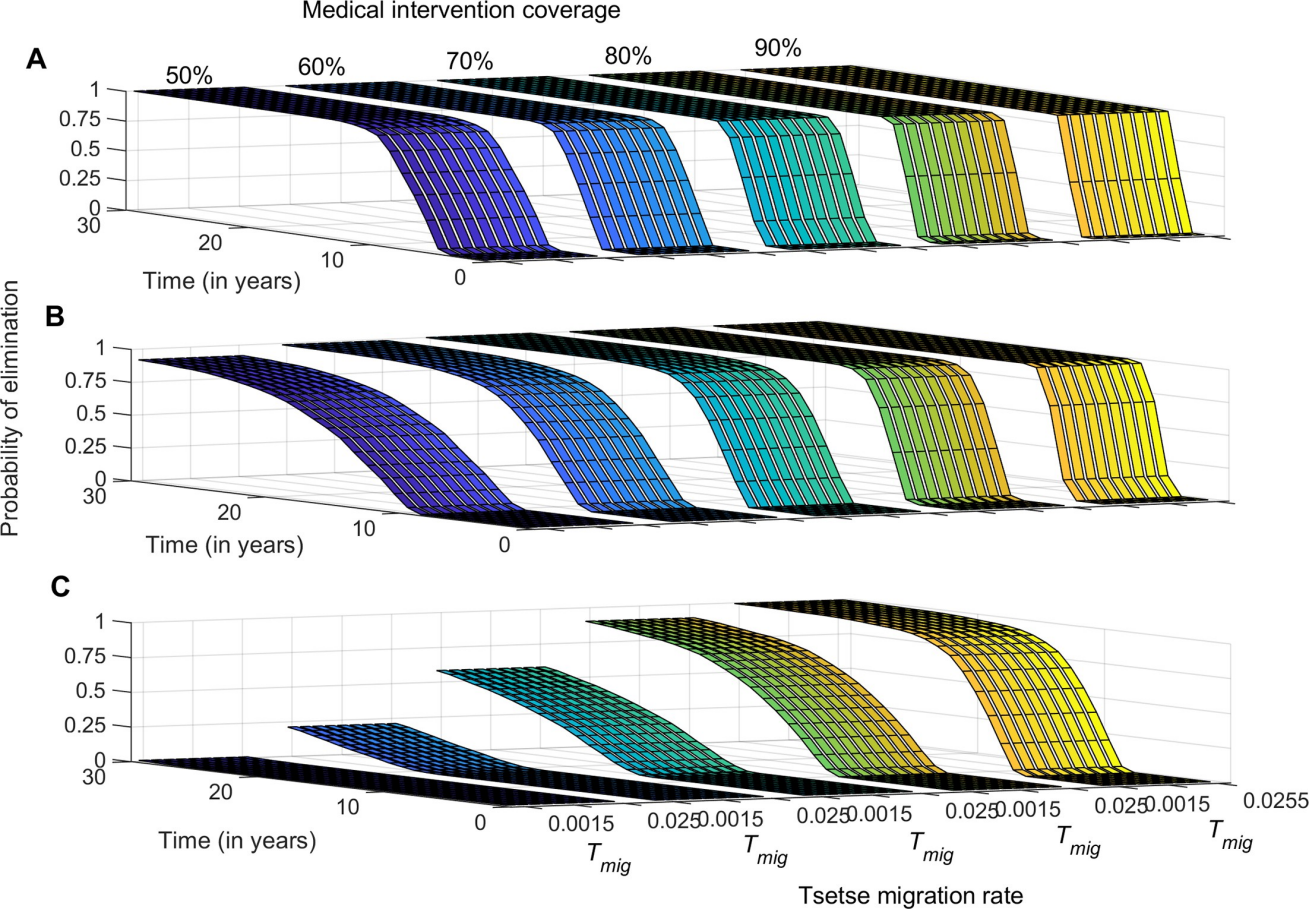
Probability of g-HAT elimination (interruption of disease transmission) over time in the absence of vector control. Probability is estimated for varying treatment coverage and tsetse migration rate. A) Low-transmission intensity settings, B) medium-transmission intensity settings, and C) high-transmission intensity settings.

Enhancing medical intervention was shown to moderately increase the probability of interrupting g-HAT transmission in low transmission settings. However, this probability decreases with increasing intensity of transmission (Fig 1). In low-transmission settings, we show that with enhanced medical intervention alone, a treatment coverage of at least 60% of all infected individuals is required to reach a 0.95 probability of elimination within 10 years (Fig 1A). In moderate transmission settings, an 80% treatment coverage is required to achieve a 0.95 probability of elimination within 10 years (Fig 1B). In high transmission intensity foci, the probability of interrupting disease transmission using medical intervention alone remains below 0.6 within 10 years of continuous treatment (Fig 1C). Tsetse migration is shown to have little to no impact on the effectiveness of medical intervention for achieving interruption of disease transmission within g-HAT foci (Fig 1).

### Medical intervention with vector control

**Fig 2:**
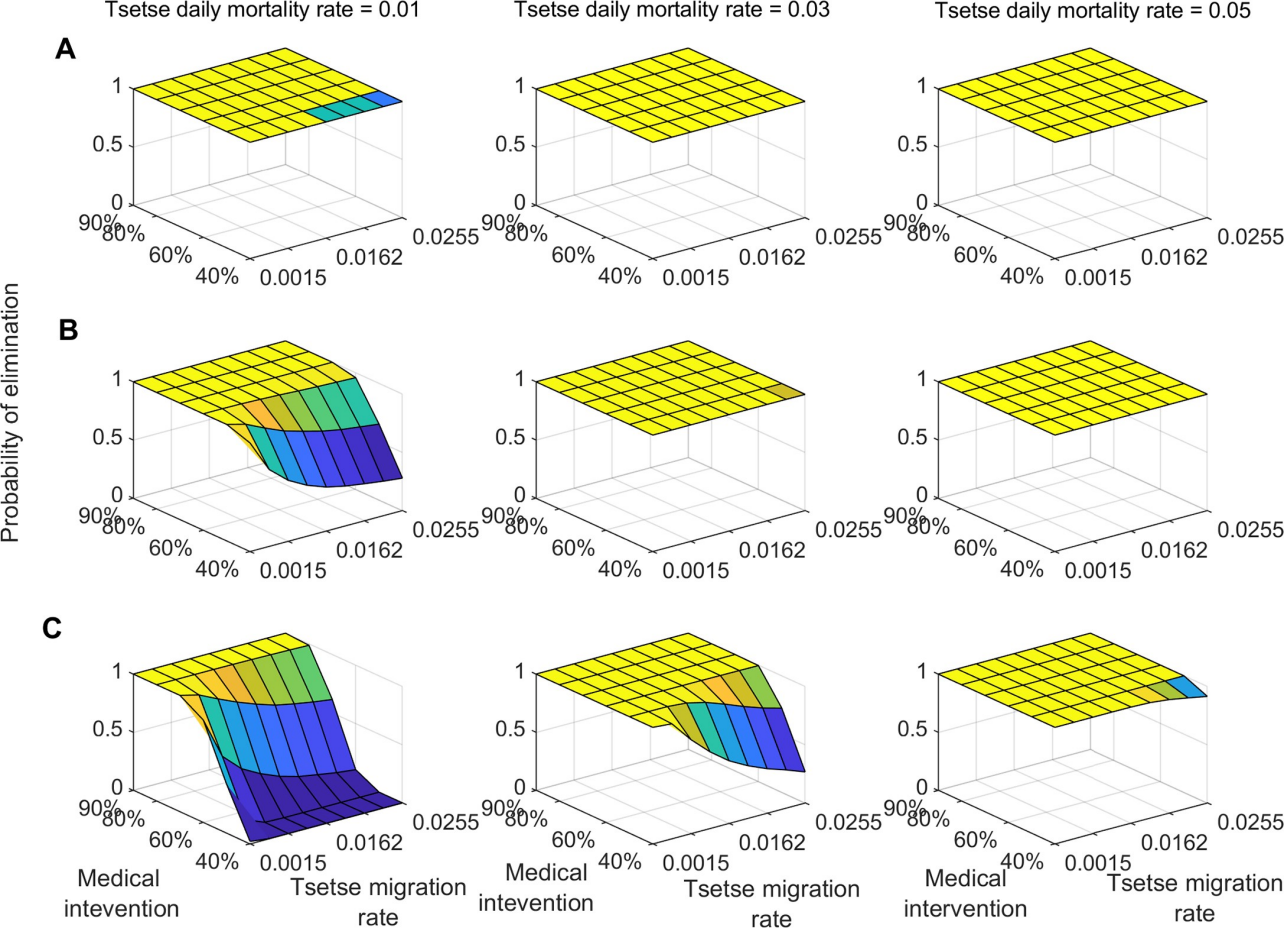
Probability of g-HAT elimination (interruption of disease transmission) at 10 year following joint initiation of enhanced medical intervention and vector control. Probability is estimated for varying value of treatment coverage and vector control induced daily mortality rate. A) Low-transmission intensity settings, B) medium-transmission intensity settings, and C) high-transmission intensity settings.

We evaluate the probability of interrupting g-HAT transmission in the three transmission intensity settings using a combination of medical intervention with vector control. Our analysis shows that in low transmission settings, a combination of medical intervention and vector control with an efficacy of 0.01 daily tsetse mortality rate is sufficient to achieve at least 0.95 probability of elimination within 10 years (Fig 2A). In these settings, tsetse migration has a marginal impact on the probability of interrupting g-HAT transmission. In moderate transmission settings, a vector control efficacy of at least 0.03 daily tsetse mortality rate is needed to achieve a 1.0 probability of g-HAT elimination within 10 years (Fig 2B). For lower vector control efficacy, the probability of interrupting disease transmission is shown to decrease with increased tsetse migration rate when medical intervention coverage is lower than 60% (Fig 2B).

**Fig 3:**
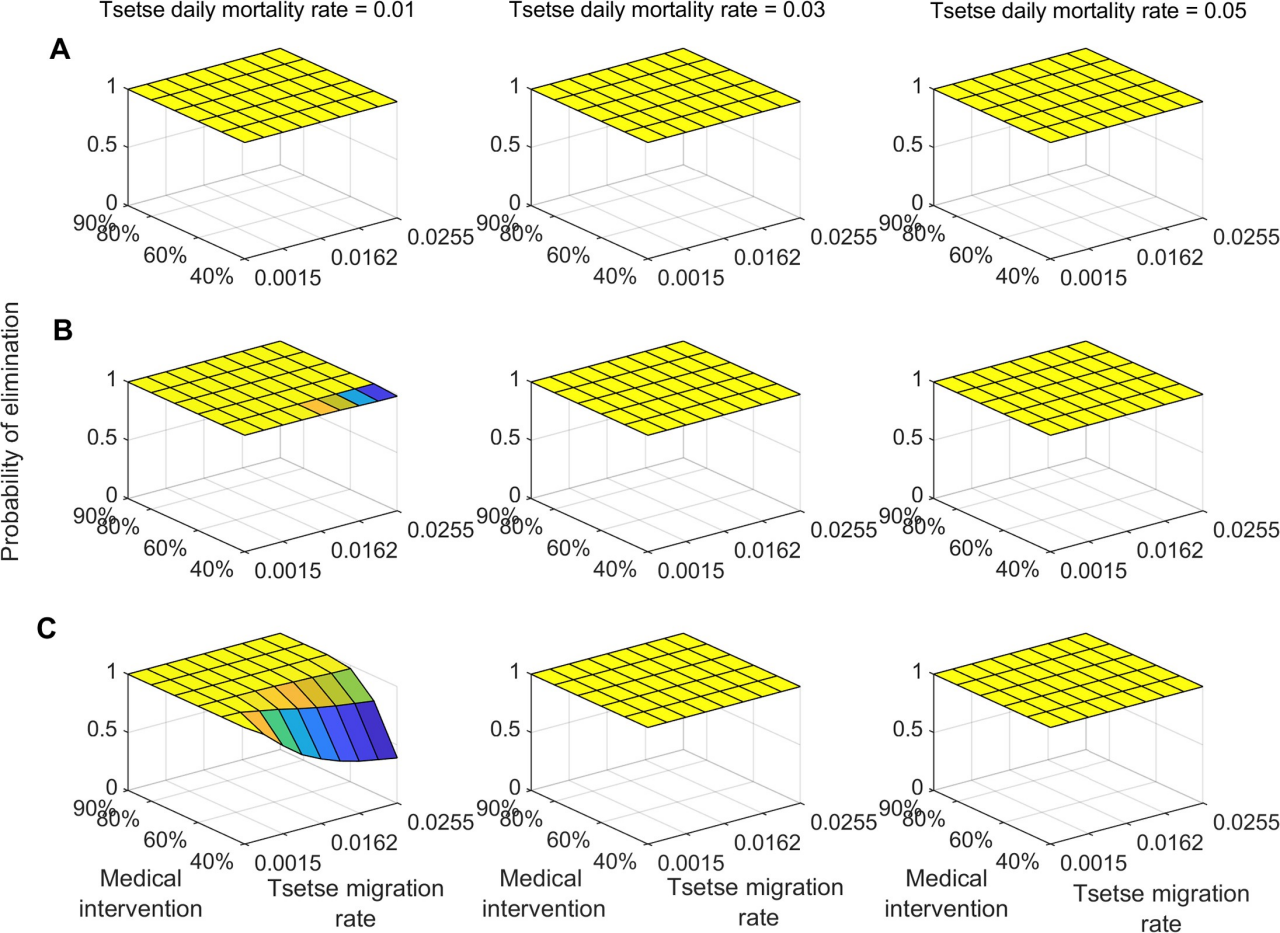
Probability of g-HAT elimination (interruption of disease transmission) at 20 year following joint initiation of enhanced medical intervention and vector control. Probability is estimated for varying value of treatment coverage and vector control induced daily mortality rate. A) Low-transmission intensity settings, B) medium-transmission intensity settings, and C) high-transmission intensity settings.

In high transmission settings, the probability of interrupting disease transmission within 10 years is shown to vary substantially with vector control efficacy and tsetse migration rate (Fig 2C). For vector control efficacy greater than 0.03 daily tsetse mortality rate, at least 60% medical intervention coverage is needed to achieve a 0.95 probability of g-HAT elimination (Fig 2C). For vector control efficacy lower than 0.03 daily mortality rate, a 0.95 probability of achieving g-HAT elimination with 10 years requires at least 80% medical intervention coverage (Fig 2C). For 20 years of continuous interventions, combining medical intervention with vector control measure of at least 0.01 daily mortality rate is sufficient to achieve a 0.95 probability of g-HAT elimination in low and moderate transmission settings (Fig 3A & 3B). In high transmission settings, the probability of achieving interruption of g-HAT transmission is shown to be greater than 0.95 for vector control efficacy of more than 0.03 daily tsetse mortality rate combined with a 60% medical intervention coverage (Fig 3C).

## Discussion

The London 2012 declarations on neglected tropical diseases identified gambiense human African trypanosomiasis (g-HAT) as a target for interruption of disease transmission by 2030. To forecast whether these elimination targets will be met and to identify feasible strategies to facilitate elimination, there has been a recent upswing in the development of g-HAT transmission models. Mathematical modelling plays a pivotal role in this endeavor as it can capture the complex and heterogeneous disease ecology of g-HAT and the nonlinear effects of g-HAT control programs. Although controlling tsetse vector populations has been used successfully to reduce g-HAT incidence in disease foci across Western and Central Africa, the presence of migration could generate re-invasion of the targeted zones by neighboring populations [42,45]. In this analysis we developed a mathematical model of g-HAT and evaluated the impact of tsetse migration on interrupting HAT transmission in endemic foci. We quantify how tsetse migration, HAT transmission intensity, and disease control together affect the probability of interrupting g-HAT transmission in a focus.

Our results indicate that achieving the WHO 2030 target will necessitate enhanced control programs including medical intervention and vector control. While a moderate scale up of medical intervention would be sufficient to curtail g-HAT transmission when used in combination with vector control in low and moderate transmission foci, achieving this gain without vector control would require much greater disease detection and treatment efforts. Regardless of improvements in case detection, active screening is highly labor intensive and requires mobile teams covering large areas of rural country. Relying on active surveillance alone to achieve elimination therefore might be infeasible.

In high transmission settings, the combined use of highly efficient vector control measures and high case detection and treatment coverage is needed to achieve a 0.95 probability of achieving the 2030 target. However, if control efforts are halted after 2030, a rebound in g-HAT cases is highly likely. Rebounds have a historical precedent: after political and economic instability caused g-HAT control efforts to falter in the 1970s, the disease rebounded in much of Western and Central Africa [46].

A limitation of our study is the use of deterministic model to evaluate disease elimination. Though such modeling approach captures the average behavior of the system, it may miss some aspects of disease transmission, especially in the context of small populations and low infection prevalence, where stochastic fade-out or take-off may play an important role. To evaluate disease elimination, we arbitrarily choose a very low threshold for annual disease incidence of 10^−7^. Using a higher threshold value will surely generate higher probability values than those obtained in this study. However, we opted for more conservative results. Future modeling studies should use stochastic simulation methods, such as Gillespie algorithm [47] or tau-leap algorithm [48], to complement their deterministic evaluation of g-HAT elimination in endemic foci.

Previous modeling studies have highlighted the importance of using vector control in combination with medical intervention for achieving interruption of HAT transmission in moderate and high transmission intensity settings [15–17]. However, these studies did not account for the potential replenishment of tsetse populations within HAT foci, through migration from neighboring population clusters. Though traps and targets increase adult tsetse mortality, which can drastically reduce tsetse populations, obtaining low tsetse density may not be enough to eliminate g-HAT in endemic foci. Our results show that the success of vector control-based programs for interrupting g-HAT transmission is highly dependent on the rate of tsetse migration, especially in high transmission intensity foci.

Tsetse migration has previously been shown to be negatively density-dependent [49]. This negative density-dependent dispersal of tsetse flies implies that immigration decreases with increased density of tsetse population in a given focus. But when population densities are low, migration is easier and tsetse flies may migrate over long-distance. Tsetse dispersal has mainly be quantified using data from release-recapture studies [50]. Estimations from these dispersal from point source have shown that tsetse dispersal follow distributions similar to bivariate normal distributions [21]. This indicates that under normal conditions, only a few flies may disperse over long distances. However, detailed empirical studies on tsetse dispersal, are needed to elucidate heterogeneity in the distribution of tsetse dispersal.

Several vector control methods have been used to control tsetse fly populations in many g-HAT endemic countries [51]. These include tools such as traps, targets, Sterile Insect Technique and aerial sprays [52]. Recently, traditional insecticide-treated targets were modified to produce “Tiny targets”, a more sustainable vector control method. These targets consist of a small square of blue cloth flanked by a similar sized piece of black netting. Tiny targets have been shown to be highly effective and more cost-effective than traps or large targets typically used for the control of g-HAT vector species [51]. These tiny targets are currently used in large-scale control programs against sleeping sickness in HAT endemic countries such as Uganda, Kenya, Chad, Guinea, Cote d’Ivoire and the Democratic Republic of Congo [8,27,41,43].

Since vector control programs aim to substantially reduce tsetse population densities, these control strategies may unintentionally unleash tsetse dispersal through negative density-dependent dispersal and subsequently cause rapid reinvasion of controlled areas from neighboring tsetse populations. Though vector control measures are effective tools for g-HAT control, a major challenge to sustaining their long-term benefits is the rebound of tsetse population following short-term control efforts. A rebound could originate from residual pockets of flies or migrants from neighboring non-targeted areas [53]. Migration between tsetse population clusters are generally facilitated by corridors of suitable habitat, such as waterways, which connect discrete patches of riverine and lacustrine habitats [53]. Tsetse reinvasion, through migration, could mitigate the long-term impact of vector control for reducing disease transmission. Therefore, identifying migration corridors into g-HAT foci and targeting them for vector control would be paramount for optimizing the effectiveness of control efforts for interrupting g-HAT transmission [25,53]. In addition to insecticide treated targets and traps, other vector control methods such as sterile insect technique (SIT) has been successfully used for tsetse control/eradication [12,54]. SIT is regarded as the technique with all necessary “qualities” for tsetse species elimination, because its efficiency increases as the density of the targeted population decreases [54,55]. However, there are a number of limitations/disadvantages to SIT: i) SIT is a costly, logistically challenging and management intensive method for tsetse species control [54,55]; ii) suppression of tsetse populations using conventional methods may be needed before SIT can be used to eradicate residual populations [55]; and iii) the efficacy of SIT for eradicating tsetse in areas with multiple species remains uncertain.

Tsetse population density varies between population clusters and fluctuates with seasonality [29]. These variations of population density, coupled with migration, affect both tsetse population dynamics and human tsetse exposure. Therefore, g-HAT transmission risk may vary with tsetse migration and seasonality. Future modeling studies should investigate the potential impact of seasonality in tsetse population densities, tsetse migration, and human-tsetse contact on the effectiveness of control measures for interrupting g-HAT transmission.

Data-driven spatially explicit models for tsetse migration can play a pivotal role for improving area-wide vector control efforts for interrupting g-HAT transmission. By accounting for spatial dispersal of tsetse flies between targeted foci and neighboring areas, such models would inform optimal density and spatial deployment of targets/traps, frequency and duration of intervention needed to prevent tsetse fly’s re-invasion and resurgence of g-HAT post disease elimination within foci. Detailed information and data on tsetse density, movement ecology, and migration rates, for the area of interest, would be needed to correctly parameterize these models. Previous modeling studies have used spatially continuous and agent-based models to evaluate the effectiveness of different control measures for reducing or eradicating tsetse flies at different spatial scales [20,25,56–58]. Other studies, statistical models of species distribution, such as logistic regression and Maxent models, have been used to optimize the deployment of insecticide-treated targets, release density of sterile tsetse males, and location of monitoring traps for a tsetse eradication campaign in Senegal [59]. Though spatial models have not yet been used to inform g-HAT elimination efforts, such an approach would be of great value to optimize control efforts. A recent study developed a landscape modeling approach that integrates genetic distances and remotely sensed environmental data to identify isolated clusters of tsetse populations [60]. This approach can be used to optimize tsetse control programs by identifying and prioritizing intervention areas (tsetse clusters) most suitable to eradication [60].

In addition to tsetse migration, other factors such as human population risk of tsetse bites and the potential presence of non-human reservoirs for g-HAT may contribute to disease persistence in endemic foci [13,34]. In our model, we subdivided the human population into high- and low-risk groups. We assumed that high- and low-risk group individuals were equally screened and treated at the same rate. Though this assumption would have minimal impact on the effectiveness of vector control efforts, it may overestimate the impact of medical intervention on the reduction of g-HAT incidence, especially if high risk groups have a lower adherence to medical intervention. In this scenario, targeted screenings among high risk groups can bolster the effectiveness of medical interventions. Our simplistic assumptions may not fully capture heterogeneity in individual risk of tsetse bites and their contribution to g-HAT transmission and control. Future models should investigate the contribution of risk behavior to disease persistence in different g-HAT incidence intensity settings. Previous modeling studies have shown that the presence of non-human animal reservoirs would reduce the effectiveness of both medical intervention and vector control for interrupting disease transmission in high transmission intensity foci [14,15,20,25,56–58]. Therefore, tsetse migration and non-human animal reservoirs may have a synergistic impact in reducing the effectiveness of vector control measures. Their combined effects would require higher medical and vector control efforts to achieve interruption of disease transmission, especially high g-HAT transmission foci.

Our study suggests that it may be impractical to achieve g-HAT elimination in moderate or high transmission settings without a combination of medical intervention and vector control. Furthermore, the success of vector control-based programs in these transmission settings is highly contingent on local tsetse migration rates. Given the importance of tsetse migration corridors to tsetse dispersal in HAT endemic areas [45], identification, targeting, and monitoring of these corridors should be undertaken in vector control programs, especially in moderate and high transmission settings. Population genetics studies have proved very useful for setting up successful vector control programs [24,25]. Such studies provide invaluable information on tsetse effective population sizes and migration rates [24,25,45]. Incorporating 1) tsetse surveillance data from vector control programs, and 2) effective population size and migration rate from population genetics studies, into simulation models would provide more accurate estimations of vector control efforts needed to achieve interruption of disease transmission. Sleeping sickness modeling would greatly benefit from having access to these data that have been collected for many tsetse fly control programs.

## Supporting information

S1 Text (DOCX)

S1 Table Definition and values of model parameters. (DOCX)

S1 Fig Posterior distributions of calibrated parameters for A) low- transmission intensity setting, B) medium-transmission intensity setting, and C) high-transmission intensity setting. The Brooks-Gelman-Rubin (BGR) method was used to monitor convergence of iterative simulations. Convergence was achieved when the upper limit of the credible interval of the BGR diagnostic statistic for a given parameter < 1.2.
(DOCX)

S2 Fig MCMC iterations for the 3 independent runs in low-transmission intensity settings. (DOCX)

S3 Fig MCMC iterations for the 3 independent runs in medium-transmission intensity settings. (DOCX)

S4 Fig MCMC iterations for the 3 independent runs in high-transmission intensity settings. (DOCX)
