# Supplementary material for: The impact of vector migration on the effectiveness of strategies to control gambiense human African trypanosomiasis
Source: PLoS Negl Trop Dis. 2019 Dec 5;13(12):e0007903. doi: 10.1371/journal.pntd.0007903 (PMC6894748; doi:10.1371/journal.pntd.0007903)
Supplement: S1 Text — (DOCX) [file pntd.0007903.s001.docx]

**S1 Text: The impact of vector migration on the effectiveness of strategies to control gambiense human African trypanosomiasis**

Martial L Ndeffo-Mbah^1,2^, Abhishek Pandey^3,4^, Katherine E Atkins^5,6,7^, Serap Aksoy^4^, Alison P Galvani^3,4^

1 Department of Veterinary Integrative Biosciences, Texas A&M College of Veterinary Medicine and Biomedical Sciences, College Station, TX, USA

2 Department of Epidemiology and Biostatistics, Texas A&M School of Public Health, College Station, TX, USA

3 Center for Infectious Disease Modeling and Analysis, Yale School of Public Health, New Haven, CT, USA

4 Department of Epidemiology and Microbial Diseases, Yale School of Public Health, New Haven, CT, USA

5 Department of Infectious Disease Epidemiology, Faculty of Epidemiology and Population Health, London School of Hygiene and Tropical Medicine, UK

6 Centre for Mathematical Modelling of Infectious Diseases, London School of Hygiene and Tropical Medicine, UK

7 Centre for Global Health Research, The Usher Institute for Population Health Sciences and Informatics, Edinburgh Medical School, The University of Edinburgh, UK

We considered three different transmission intensity settings defined as reporting 1 to 10 cases per 100000 inhabitants annually for low incidence settings, 10 to 100 cases per 100000 inhabitants annually for moderate incidence, and 100 to 1000 cases per 100000 inhabitants annually for high incidence. Tsetse migration rate,$T_{mig}$, was informed by empirical estimates of 0.05 to 0.85 per tsetse generation [[31,32]](https://paperpile.com/c/bPz99N/zR7y+0Tie). A tsetse generation was estimated to be equal to ${1/\mu}_{V}=33.3 days$. The daily migration rate was estimated by converting the empirical per generation estimate to daily estimate by dividing it by 33.3 days/generation. We set the base value of migration rate to be equal to 0.05*$\mu_{V}.$We model migration as a density-dependent function following previous modeling studies [[18,19]](https://paperpile.com/c/bPz99N/urUk+J5qH). Consistent with empirical studies, we assumed a negative density-dependent migration of tsetse population [[49]](https://paperpile.com/c/bPz99N/fvhB). Under this assumption, immigration into the tsetse population decreases with density. The model is expressed by the following system of differential equations:

$dV_{P}=B_{V}N_{V}-(\varepsilon_{P}+\mu_{P})V_{P}$ (1.1)

$dV_{S}=\varepsilon_{P}V_{P}-\alpha V_{S}-\mu_{V}(1+N_{V}/\kappa)V_{S}+T_{mig}(N_{V}^{*}-N_{V})g(V_{S})-\mu_{c}V_{S}$ (1.2)

$dV_{E}=\alpha f_{H}\beta_{HV}\left( \frac{c_{h}(H_{Ih}^{1}+H_{Ih}^{2})}{c_{h}N_{Hh}+c_{l}N_{Hl}}+\frac{c_{l}(H_{Il}^{1}+H_{Il}^{2})}{c_{h}N_{Hh}+c_{l}N_{Hl}} \right)V_{s}-{(\tau}_{V}+\mu_{V}(1+N_{V}/\kappa)+\mu_{c})V_{E}+T_{mig}(N_{V}^{*}-N_{V})g(V_{E})$ (1.3)

${dV_{I}=\tau}_{V}V_{E}-\mu_{V}(1+N_{V}/\kappa)V_{I}+T_{mig}(N_{V}^{*}-N_{V})g(V_{I}) -\mu_{c}V_{I}$ (1.4)

$dV_{R}= \alpha\left( 1-f_{H}\beta_{HV}\left( \frac{c_{h}(H_{Ih}^{1}+H_{Ih}^{2})}{c_{h}N_{Hh}+c_{l}N_{Hl}}+\frac{c_{l}(H_{Il}^{1}+H_{Il}^{2})}{c_{h}N_{Hh}+c_{l}N_{Hl}} \right) \right)V_{s}-(\mu_{V}\left( 1+\frac{N_{V}}{\kappa} \right){+\mu_{c})V}_{R}+T_{mig}\left( N_{V}^{*}-N_{V} \right)g\left( V_{R} \right)$(1.5)

$V_{P}$represents pupae of tsetse population, $V_{S}$ represents susceptible tsetse population, $V_{E}$exposed tsetse population,$V_{I}$infected tsetse population,$V_{R}$recovered tsetse population, with $N_{V}=V_{S}+V_{E}+V_{I}+V_{R}$ the total population of adult tsetse. The notation *dV* represents the time dependent derivative of *V*.

Adult mortality is density-dependent, and increases linearly as a function of $N_{V}$. Setting the adult density at equilibrium as${N_{V}^{*}=n}_{HV}N_{H}$, where $n_{HV}$is the relative density of tsetse flies per host, yields $\kappa=N_{V}^{*}(\frac{\mu_{V}(\varepsilon_{P}+\mu_{P})}{\varepsilon_{P}B_{V}-\mu_{V}(\varepsilon_{P}+\mu_{P})})$, and $B_{V}>\mu_{V}(1+\frac{\mu_{P}}{\varepsilon_{P}})$ensures adult mortality grows with adult density at baseline. $\mu_{c}$is tsetse mortality rate due to vector control and was set to be equal to 0 at baseline.

The migration function $g(V_{\cdot})$is defined as follows: if $N_{V}>N_{V}^{*}$ then $g(V_{\cdot})=\frac{V_{\cdot}}{N_{V}}$; here tsetse migrate out to neighbouring HAT-free area. if $N_{V}<N_{V}^{*}$ then $g(V_{S})= \mu_{V}(1+N_{V}^{*}/\kappa)/({\alpha+\mu}_{V}(1+N_{V}^{*}/\kappa)), g(V_{E})=0, g(V_{I})=0, g(V_{R})=\alpha/({\alpha+\mu}_{V}(1+N_{V}^{*}/\kappa));$here tsetse migrate into the population from neighboring HAT-free area. By assuming that tsetse in the HAT-free areas are at equilibrium and that the HAT foci and neighboring HAT-free area have the same carrying capacity, the proportion of susceptible and removed tsetse are equal to $\mu_{V}(1+N_{V}^{*}/\kappa)/({\alpha+\mu}_{V}(1+N_{V}^{*}/\kappa))$ and $\alpha/({\alpha+\mu}_{V}(1+N_{V}^{*}/\kappa))$, respectively.

Human infection dynamics is modelled by the following system of differential equations:

${dH}_{Sj}=B_{H}(N_{H})P_{j}+\delta_{H}^{T} H_{Tj} -d_{H}H_{Sj}-\alpha f_{H}\beta_{VH}c_{j}V_{I}\frac{c_{j}H_{Sj}}{c_{h}N_{Hh}+c_{l}N_{Hl}}$ (2.1)

${dH}_{Ej}=\alpha f_{H}\beta_{VH}c_{j}V_{I}\frac{c_{j}H_{Sj}}{c_{h}N_{Hh}+c_{l}N_{Hl}}-\left( \tau_{H}+d_{H} \right)H_{Ej}$ (2.2)

${dH}_{Ij}^{1}=\tau_{H}H_{Ej}-\left( \gamma_{H}^{1}+d_{H}+{\varsigma\gamma}_{T} \right)H_{Ij}^{1}$ (2.3)

${dH}_{Ij}^{2}=\gamma_{H}^{1}H_{Ij}^{1}-\left( \gamma_{H}^{2}+d_{H}+{\varsigma\gamma}_{T} \right)H_{Ij}^{2}$ (2.4)

${dH}_{Tj}={\varsigma\gamma}_{T}\left( H_{Ij}^{1}+H_{Ij}^{2} \right)-\left( \delta_{H}^{T}+d_{H} \right) H_{Tj}$ (2.5)

$H_{Sj}$represents susceptible human of risk-group *j*, $H_{Ej}$exposed human of risk-group *j*,$H_{Ij}^{1}$infected human in stage 1 of risk-group j, $H_{Ij}^{2}$infected human in stage 2 of risk-group *j*, $H_{Tj}$treated human of risk-group *j*. $j\in\left\{ l,h \right\}$, with $l$ = low risk-group and $h$ = high risk-group. $c_{h}$is the relative contact of tsetse with high risk group; $c_{l}$is the relative contact of tsetse with low risk group, with $c_{l}=1$. $N_{Hj}$the total human population size in risk-group *j*, with $N_{Hj}=H_{Sj}+H_{Ej}+H_{Ij}^{1}+H_{Ij}^{2}+H_{Tj}$being the total population of humans in risk-group j. The total population size $N_{H}=N_{Hh}+N_{Hl}$. For simplicity, we set $N_{H}=$1. $P_{j}$is the proportion of the population in risk-group *j*, with $P_{h}+P_{l}=1$. To keep the total human population constant, we set $B_{H}\left( N_{H} \right)=\sum_{j} \left( d_{H}N_{Hj}+\gamma_{H}^{2}H_{Ij}^{2} \right).$We relate the percentage of screened individuals *r* to the screen and treat rate $\gamma_{T}$as follow $\gamma_{T}=-ln(1-r/100)/365$[[18]](https://paperpile.com/c/bPz99N/urUk). The baseline screen and treat coverage was set to $r=40\%$. The model was calibrated to constrain incidence within specific ranges using a Bayesian MCMC approach with metropolis-hastings algorithm [[37,38]](https://paperpile.com/c/bPz99N/ozQL+xvsi) with a uniform likelihood function. We ran 3 independent MCMC runs. Each run consisted of a 10,000 MCMC iterations followed by 500,000 iterations. For each run, the starting point was generated randomly. The prior distributions of calibrated parameters are given in Table S1, and the posterior distributions are shown in Fig S1.
