# Supplementary material for: The impact of vector migration on the effectiveness of strategies to control gambiense human African trypanosomiasis
Source: PLoS Negl Trop Dis. 2019 Dec 5;13(12):e0007903. doi: 10.1371/journal.pntd.0007903 (PMC6894748; doi:10.1371/journal.pntd.0007903)
Supplement: S1 Table — (DOCX) [file pntd.0007903.s002.docx]

S1 Table: Definition and values of model parameters

| Parameter | Definition | Values/Priors* | References |
| --- | --- | --- | --- |
| Fixed parameters | | | |
| B*_V_* | Pupal deposit rate | 0.0505 days^-1^ | [[17]](https://paperpile.com/c/bPz99N/lm1U) |
| $\varepsilon$*_P_* | Pupal maturation rate | 0.022 days^-1^ | [[61]](https://paperpile.com/c/bPz99N/OVPV) |
| $\mu$*_P_* | Pupal death rate | 0.006 days^-1^ | [[61,62]](https://paperpile.com/c/bPz99N/OVPV+N0Ol) |
| $\mu$*_V_* | Baseline tsetse mortality rate | 0.03 days^-1^ | [[29]](https://paperpile.com/c/bPz99N/uTF6) |
| $\alpha$ | Tsetse biting rate | 0.333 days^-1^ | [[29]](https://paperpile.com/c/bPz99N/uTF6) |
| $\tau$*_V_* | Incubation rate in tsetse | 0.034 days^-1^ | [[63,64]](https://paperpile.com/c/bPz99N/u7iy+Bgu5) |
| $\tau$*_H_* | Incubation rate in humans | 0.0833 days^-1^ | [[29]](https://paperpile.com/c/bPz99N/uTF6) |
| $\gamma$*_H1_* | Stage 1 progression rate | 1/526 days^-1^ | [[65]](https://paperpile.com/c/bPz99N/Uu1s) |
| $\gamma$*_H2_* | Stage 2 progression rate | 1/251 days^-1^ | [[65]](https://paperpile.com/c/bPz99N/Uu1s) |
| $\delta_{H}^{T}$ | Treatment immunity loss rate | 0.1 days^-1^ | [[11]](https://paperpile.com/c/bPz99N/PraR) |
| $d$*_H_* | Human natural death rate | 4.66x10^-5^ days^-1^ | [[66]](https://paperpile.com/c/bPz99N/PRL3) |
| $\gamma_{T}$ | Baseline screen and treat rate | 0.0014 days^-1^ | [[35,36]](https://paperpile.com/c/bPz99N/39gu+E6mx) |
| $\varsigma$ | Efficacy of treatment | 0.95 | [[67,68]](https://paperpile.com/c/bPz99N/3QuP+cB9g) |
| *f_H_* | Proportion of bites on humans | 0.38 | [[15,69]](https://paperpile.com/c/bPz99N/ylyY+VpTv) |
| $T_{mig}$ | Tsetse migration rate | 0.0015 - 0.0255 days^-1^ | [[31,32]](https://paperpile.com/c/bPz99N/zR7y+0Tie) |
| $\mu$*_c_* | Vector control induced mortality rate | 0.01 - 0.08 days^-1^ | [[41]](https://paperpile.com/c/bPz99N/FvQh) |
| Fitted parameters | | | |
| $\beta$*_HV_* | Probability of tsetse infection per bite | U(0.01,0.14) | [[18,29,70]](https://paperpile.com/c/bPz99N/uTF6+urUk+oJhM) |
| $\beta$*_VH_* | Probability of human infection per bite | U(0.05,0.62) | [[18,29,70,71]](https://paperpile.com/c/bPz99N/uTF6+urUk+oJhM+t5gA) |
| *n_HV_* | Relative density of vectors per host | U(0,10) |  |
| *P_h_* | Proportion of population in high risk group | U(0,0.5) |  |
| *P_l_* | Proportion of population in low risk group | 1-*P_h_* |  |
| *c_h_* | Relative contact of tsetse with high risk group | U(1,5) |  |

* U(a,b) - Uniform distribution
