# Supplementary material for: The impact of vector migration on the effectiveness of strategies to control gambiense human African trypanosomiasis
Source: PLoS Negl Trop Dis. 2019 Dec 5;13(12):e0007903. doi: 10.1371/journal.pntd.0007903 (PMC6894748; doi:10.1371/journal.pntd.0007903)

S1 Fig. Posterior distributions of calibrated parameters for A) low- transmission intensity setting, B) medium-transmission intensity setting, and C) high-transmission intensity setting. The Brooks-Gelman-Rubin (BGR) method was used to monitor convergence of iterative simulations. Convergence was achieved when the upper limit of the credible interval of the BGR diagnostic statistic for a given parameter < 1.2.


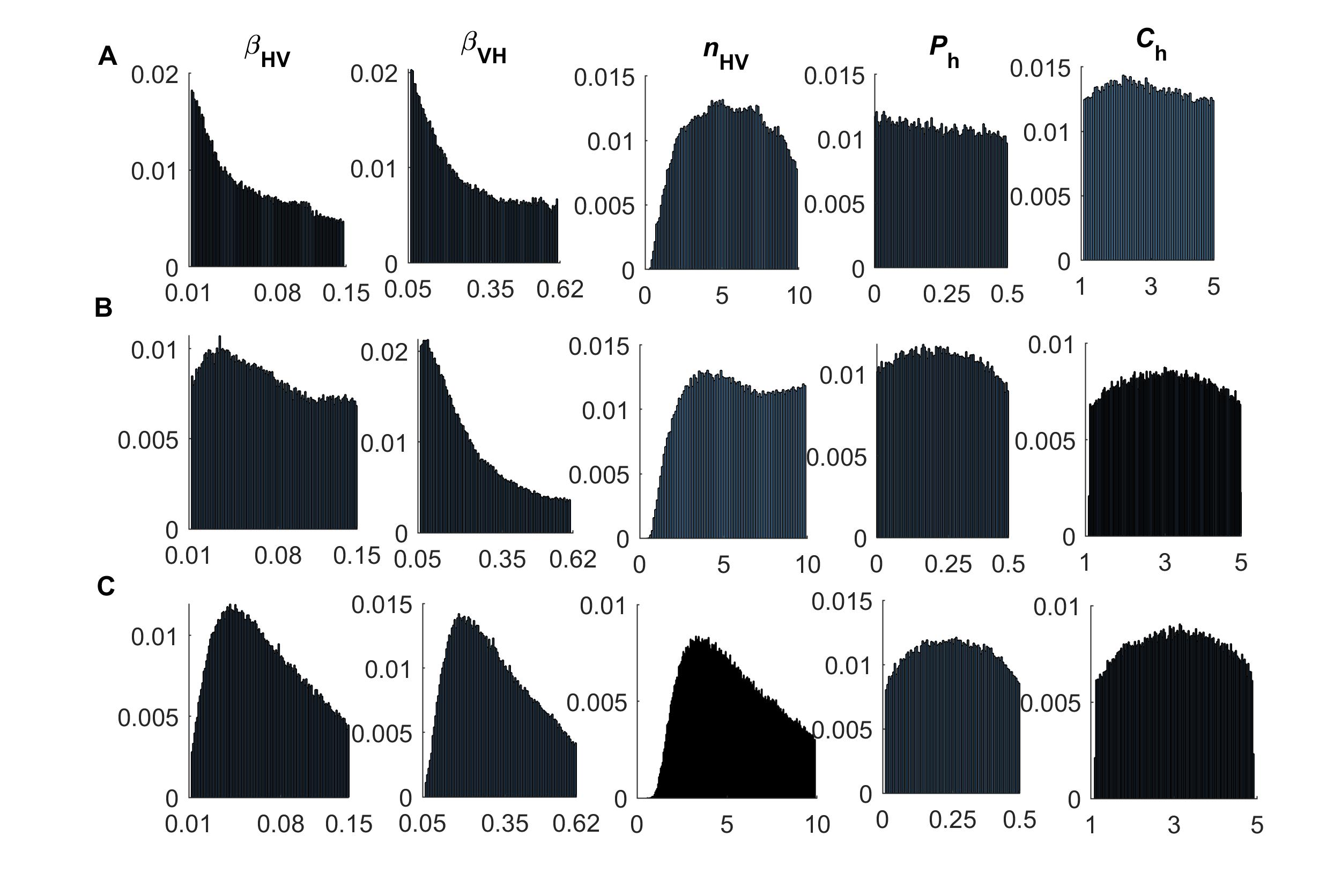

Supplement: S1 Fig — Posterior distributions of calibrated parameters for A) low- transmission intensity setting, B) medium-transmission intensity setting, and C) high-transmission intensity setting. The Brooks-Gelman-Rubin (BGR) method was used to monitor convergence of iterative simulations. Convergence was achieved when the upper limit of the credible interval of the BGR diagnostic statistic for a given parameter < 1.2. (DOCX) [file pntd.0007903.s003.docx]
