# Supplementary figures and images for: The impact of vector migration on the effectiveness of strategies to control gambiense human African trypanosomiasis
Source: PLoS Negl Trop Dis. 2019 Dec 5;13(12):e0007903. doi: 10.1371/journal.pntd.0007903 (PMC6894748; doi:10.1371/journal.pntd.0007903)

S2 Fig. MCMC iterations for the 3 independent runs in low-transmission intensity settings.


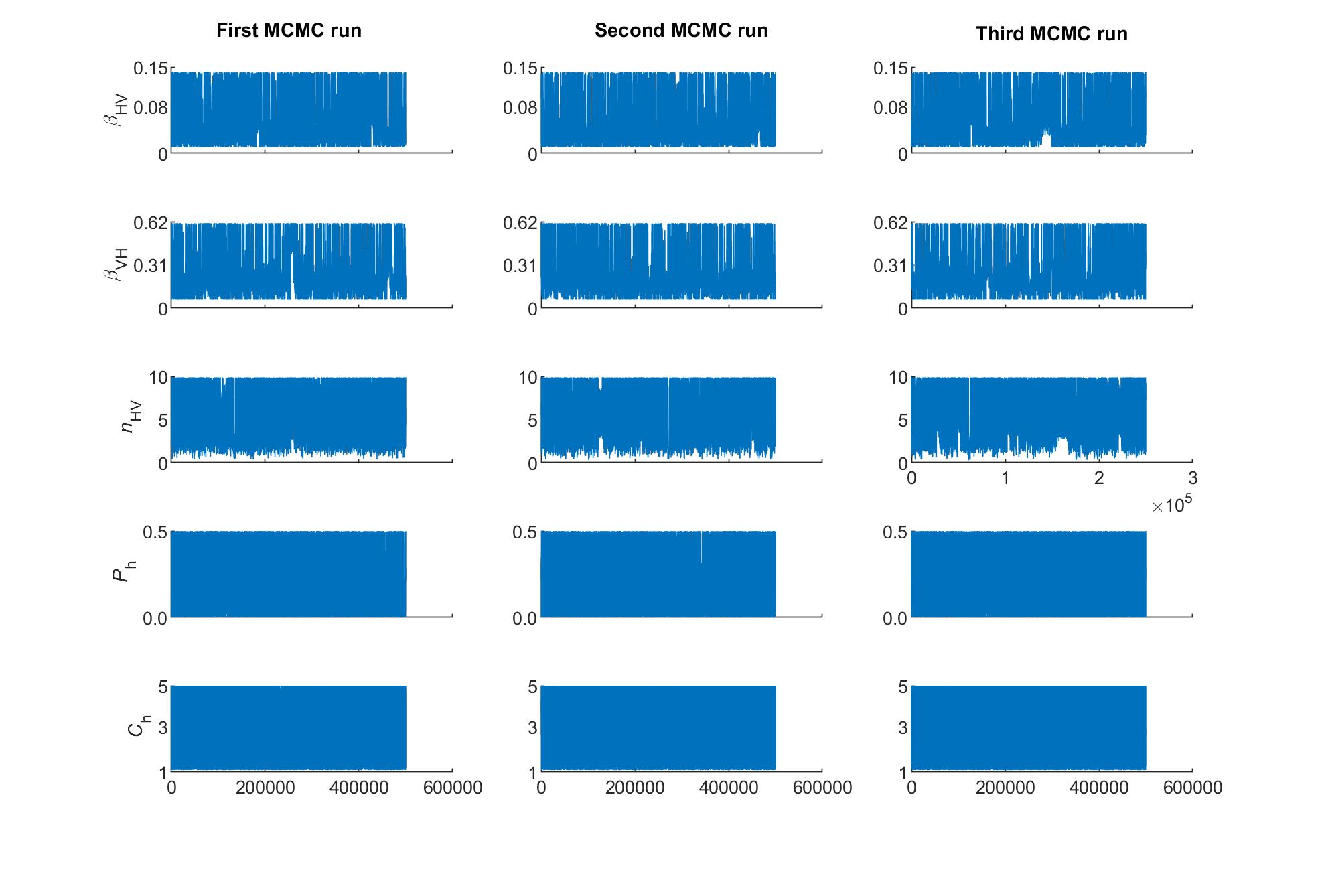

Supplement: S2 Fig — (DOCX) [file pntd.0007903.s004.docx]

S3 Fig. MCMC iterations for the 3 independent runs in medium-transmission intensity settings.


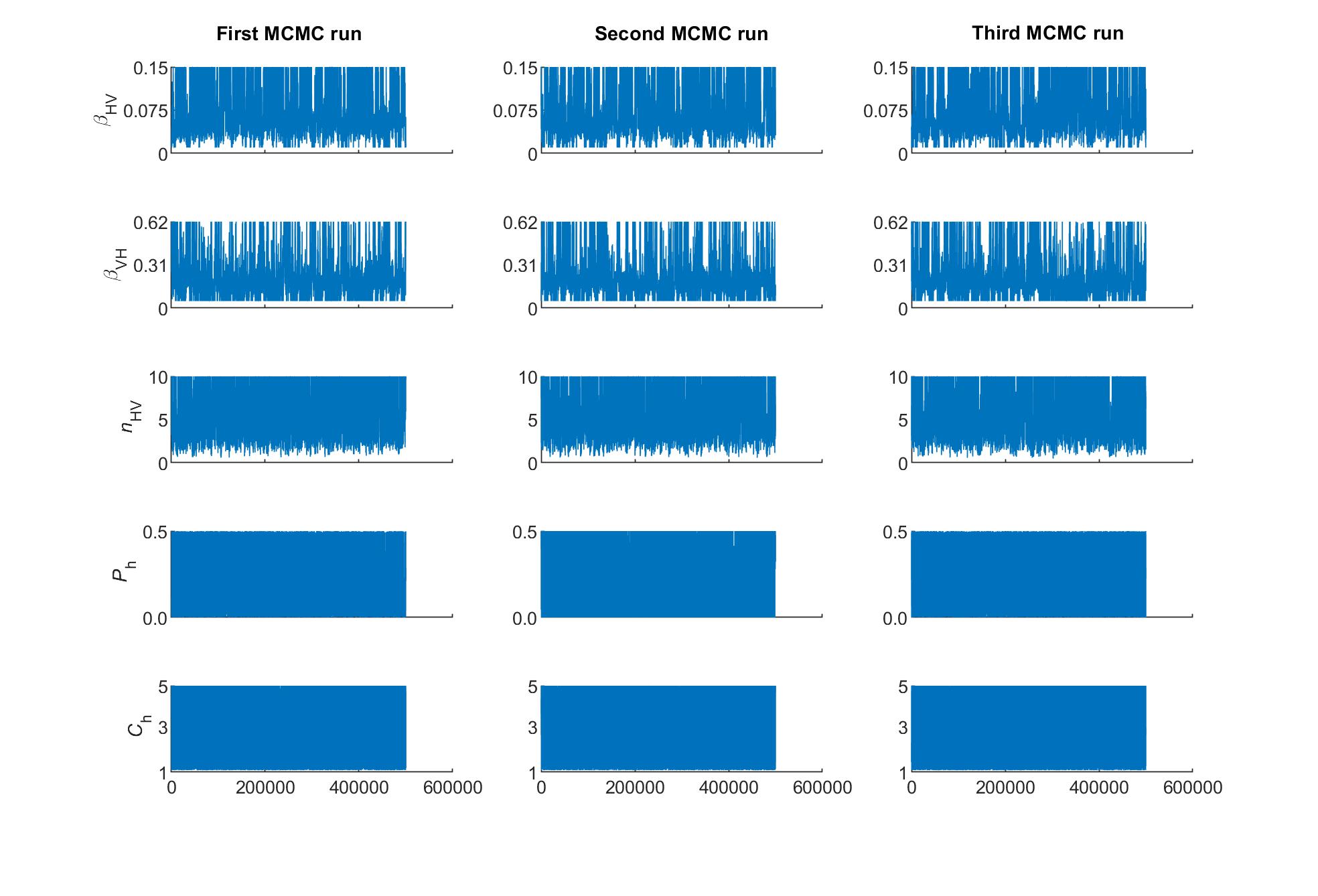

Supplement: S3 Fig — (DOCX) [file pntd.0007903.s005.docx]

S4 Fig. MCMC iterations for the 3 independent runs in high-transmission intensity settings.


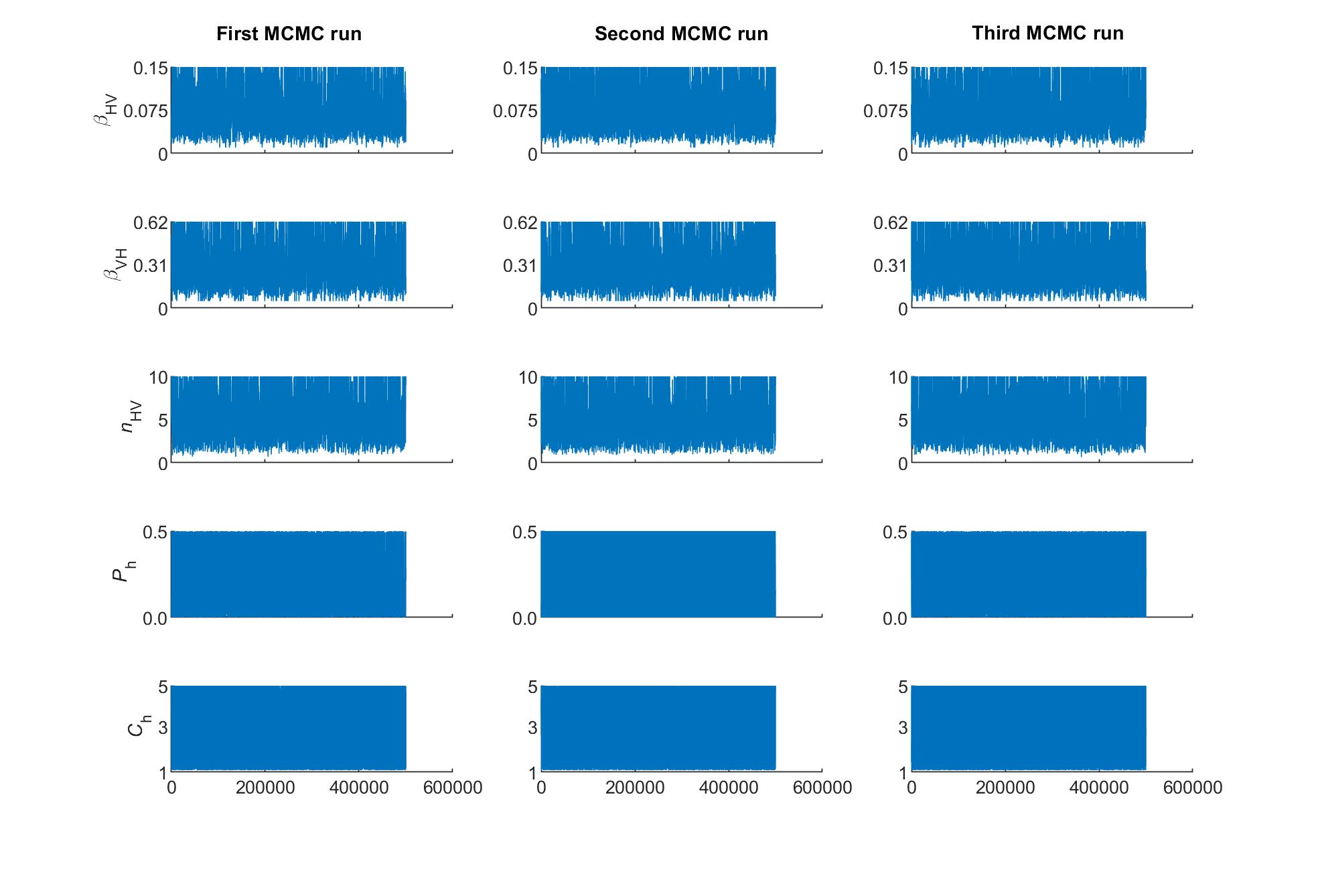

Supplement: S4 Fig — (DOCX) [file pntd.0007903.s006.docx]
